# Evaluation of Buccolingual Inclinations of First Molars Among Different Vertical Facial Patterns in Class II Division I Subjects

**DOI:** 10.7759/cureus.63724

**Published:** 2024-07-03

**Authors:** Bilal F Mahmoud, Ali B Barri, Fadi Khalil

**Affiliations:** 1 Orthodontics and Dentofacial Orthopedics, Tishreen University, Latakia, SYR; 2 Orthodontics, Private Practice, Doha, QAT; 3 Orthodontics, Tishreen University, Latakia, SYR

**Keywords:** cbct, class ii, vertical facial pattern, buccolingual molar inclination, 3d cephalometrics

## Abstract

Background

Knowing the characteristics of vertical patterns is crucial to provide the best orthodontic treatment. Cone beam computed tomography (CBCT) offers a valuable tool for evaluating true buccolingual inclinations. The current study investigates the buccolingual inclination of first molars in adult subjects with different vertical facial patterns.

Methods

CBCT scans of 66 adult patients (31 males, 35 females) with a mean age of 31.6 years (SD: 6.4 years) exhibiting skeletal class II division I maxillomandibular relationships were employed. Participants were categorized into three groups based on linear and angular measurements: normodivergent group (n=22), hypodivergent group (n=22), and hyperdivergent group (n=22). The independent samples t-test and Mann-Whitney U-test were conducted to investigate statistical differences in terms of buccolingual inclination between the three vertical patterns.

Results

Statistically significant differences were observed in the buccolingual inclinations of both maxillary and mandibular first molars in the hypodivergent group compared to the other groups (p<0.05).

Conclusions

In patients with class II division I sagittal relationships, the buccolingual inclinations of the first molars exhibit similarities between normodivergent and hyperdivergent groups. However, these inclinations differ significantly in hypodivergent adult subjects.

## Introduction

Orthodontic treatment planning requires consideration of patient-specific characteristics, including age, gender, and ethnicity. The facial pattern and its clinical features are an additional crucial component [1]. The facial pattern influences the selection of anchorage type, the prediction of craniofacial growth, and the overall goals of treatment [2]. These goals encompass dental alignment within the arches, correction of malocclusion to achieve a stable dental occlusion, and optimization of both function and aesthetics [3]. To achieve these objectives, the maxillary dentoalveolar bone must possess sufficient transverse dimension, and the posterior teeth must exhibit optimal buccolingual inclinations [4]. When observed frontally, the maxillary posterior occlusal surfaces form the convexity, and the mandibular posterior occlusal surfaces form the concavity of the occlusal plane, which normally forms a curve [5]. Wilson's curve is the name given to this occlusal curve in the coronal plane [6]. The natural curvature of the teeth is thought to allow the posterior teeth inclination to line up with the orientation and inward pull of the medial pterygoid muscle during contraction [7]. This provides optimal resistance to masticatory forces, facilitates access to food for efficient chewing, and ensures effective cuspal contact engagement [8,9].

Numerous research investigations addressing the inclinations of the posterior teeth have frequently divided their participants into groups based on sagittal [1,10-12] or vertical [2,13-17] skeletal features. Conversely, certain studies have examined inclinations without explicitly determining sagittal or vertical classification [5-7,18,19]. The majority of earlier studies have only used model casts to evaluate buccolingual inclination based on tooth crowns [1,10-13,18]. Research by Ross et al. indicated that there were no statistically significant differences among different facial patterns in terms of molar inclination [13]. Additionally, Janson et al. found that the buccal inclination of the maxillary posterior teeth was significantly greater in the hyperdivergent group [1]. Furthermore, Shu et al. compared groups based on sagittal classification and found that class II division I subjects showed more lingually inclined maxillary molars in comparison to class I subjects [10]. The limits of utilizing cast models because of crown morphology variances should be noted [6]. Hence, researchers sought the development of novel methods that would enable the visualization of both crown and root structures for an accurate assessment of tooth inclination. The development of three-dimensional imaging methods, including computed tomography (CT) and cone beam computed tomography (CBCT), allowed for the fulfillment of this requirement, which empowers practitioners to visualize and measure the true inclination of teeth [2,4-7,14-17,19,20].

Tsunori et al. conducted a study investigating the mandibular buccolingual inclinations in hyperdivergent and hypodivergent individuals. Their findings indicated that individuals with hyperdivergent facial patterns exhibited a significantly greater buccal inclination of posterior teeth compared to their hypodivergent counterparts [2]. Golshah et al. focused on the relationship between sagittal classification and molars' buccolingual inclinations. Their results demonstrated that in class II sagittal patterns, maxillary molars exhibited a reduced inclination, while mandibular molars exhibited an increased inclination [3]. Nonetheless, in a study involving individuals with class I sagittal relationships who were classified as normodivergent, hyperdivergent, and hypodivergent, Eraydin et al. reported opposing results. The buccolingual inclinations of the mandibular and maxillary molars in each of the three patterns did not significantly differ, according to their findings [4].

The results' diversity in the literature, predominantly attributable to the disparate patient classification being based on either vertical or sagittal features, has posed a challenge in ascertaining the characteristic inclinations of the posterior teeth in a given patient. Consequently, the primary objective of this investigation was to assess the buccolingual inclinations of first molars, with a specific focus on skeletal class II division I patients exhibiting varying vertical facial patterns.

## Materials and methods

The study was approved by the Institutional Review Board of Tishreen University (approval number: 4338). A power analysis was conducted using α=0.05 and power=0.8, indicating that a sample size of n=66 would provide sufficient statistical power. Archived CBCT scans from 2014 to 2022 at the Faculty of Dentistry, Tishreen University, formed the sample for this retrospective investigation. Eligible subjects met the following inclusion criteria: age between 18 and 40 years, class II division I maxillomandibular relationship, absence of diagnosed systemic diseases, craniofacial dysmorphology, impacted or missing teeth, periodontal disease, facial asymmetries (more than 2 mm deviation of ME), or cleft lip or palate.

CBCT scans were acquired using the following parameters: 85 kVp, 15 mA, 40 second exposure time, 3.3 mm focal spot, and 0.090 mm voxel size. Images were generated using a Scanora 3D CBCT Soredex unit (Tuusula, Finland) and stored in Digital Imaging and Communications in Medicine (DICOM) format.

Sixty-six CBCT scans with a number of males (n=31, 46.96%) and females (n=35, 53.03%) were employed. Three-dimensional cephalometric analyses were performed on CBCT data to ascertain the subject's sagittal and vertical skeletal features. The sample included CBCT scans of class II individuals with an ANB angle larger than 4° [21] and an upper incisor angle with the Frankfurt (FH) plane of 110° or greater [22]. Angular and linear measurements were used to determine the vertical growth pattern. The sella-nasion to gonion-menton (S-N/Go-Me) angle was utilized: S-N/Go-Me angles of less than 30.5°, between 30.5° and 35.5°, and greater than 35.5°, respectively, indicated hypodivergence, normodivergence, and hyperdivergence [23]. The S-Go/N-Me ratio was also used: a ratio of less than 61%, between 61% and 69%, and greater than 69%, respectively, indicated hyperdivergence, normodivergence, and hypodivergence [24]. Any subject who did not fulfill these requirements was excluded. Eventually, 66 patients' CBCT scans were included in the study, with 22 subjects in each group. The distribution of patients across the three groups and statistics for the measurements that were the basis for classification between groups are presented in Table 1.

**Table 1:** Numbers of females and males in each group. Statistics for the measurements that were the basis for classification between groups. Values are presented as number (N) or percentage (%) or mean±standard deviation. ANB: the angle formed between points A, N, and B; S N-Go Me: the angle formed between the line (S-N) and the line (Go-Me); S-Go/N-Me: the ratio of the length of the line segment S-Go to the length of the line segment N-Me; U1: the axis of the upper central incisors; FH: Frankfurt plane; U1-FH: the angle formed between the axis of the upper central incisors and the Frankfurt plane

Basis for classification		Hyperdivergent	Normodivergent	Hypodivergent
Gender	Male	8 (36.4%)	8 (36.4%)	15 (68.3%)
	Female	14 (63.6%)	14 (63.6%)	7 (31.8%)
ANB (degree)		7.41°±2.29°	6.23°±1.84°	6.42°±1.23°
S N-GO Me (degree)		42.27°±4.58°	33.66°±1.75°	27.31°±2.82°
S-Go/N-Me (%)		57%±2.45	65%±3.22	71%±1.56
U1-FH (degree)		117.77°±2.34°	114.56°±1.75°	115.89°±1.43°

The OnDemand program version 1.0.10.7462 (Cybermed Inc., Seoul, Korea) was utilized to analyze the radiographs. The nasion (N) was selected as the origin of the three coordinate planes (X, Y, Z). The orbital plane is established by the right and left orbitales (Or) and the left porion (Po). The horizontal plane (X) is defined as the plane parallel to the orbital plane and passing through N. The midsagittal plane (Y) is defined as the plane perpendicular to the horizontal plane, passing through N and the anterior nasal spines (ANS). Finally, the frontal plane (Z) is perpendicular to both the horizontal and midsagittal planes, passing through N. Second, a custom analysis was created within the program's 3D Ceph section, and code was input to pinpoint the following anatomical landmarks (Table 2).

**Table 2:** The landmarks that were determined for each cone beam computed tomography scan. N: nasion, S: sella, Po: porion, ANS: anterior nasal spine, Me: menton, Go: gonion, L or: left orbitale, R or: right orbitale

Landmark	Definition of the landmark
Nasion	The junction between the nasal and frontonasal sutures
S	The center of the sella turcica on the midsagittal plane
Po	The most superior point on the upper rim of the external auditory meatus
A	The deepest point between the anterior nasal spine and prosthion at the midsagittal plane
B	The deepest point between the pogonion and the alveolus of the lower incisors on the midsagittal plane
ANS	The most anterior point on the floor of the nose
Me	The lowermost point on the symphysis menti on the midsagittal plane
Go	The midway between the lowermost point on the posterior border of the ramus and the most posterior point on the lower border of the mandible
R or	The most inferior point on the lower rim of the orbit
L or	The most inferior point on the lower rim of the orbit
Crown point	A point within the level of the tooth crown that was used to determine the longitudinal axis of the molar
Root point	A point within the plane of the root bifurcation that was used to determine the longitudinal axis of the molar

**Figure 1:**
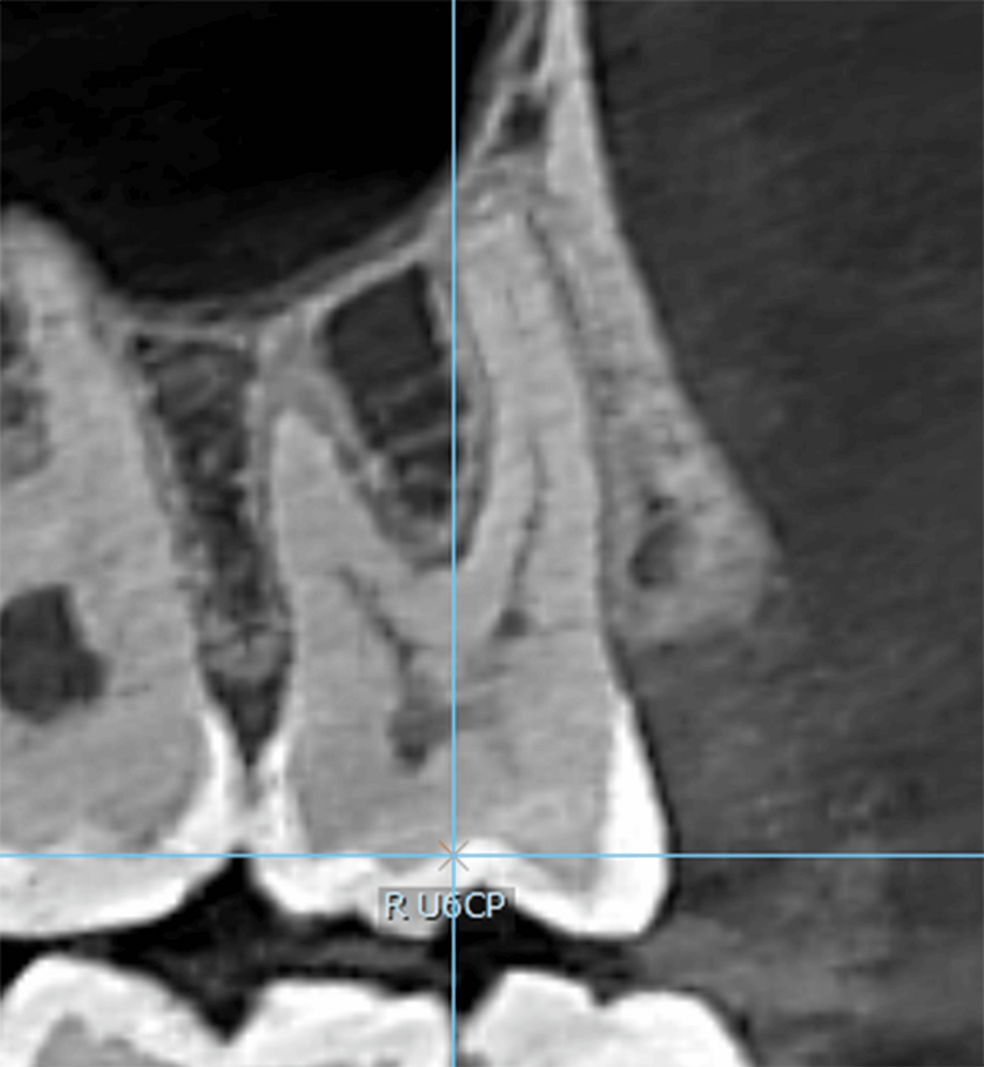
The axial axis passes through the cusps. R U6CP: right upper first molar crown point

**Figure 2:**
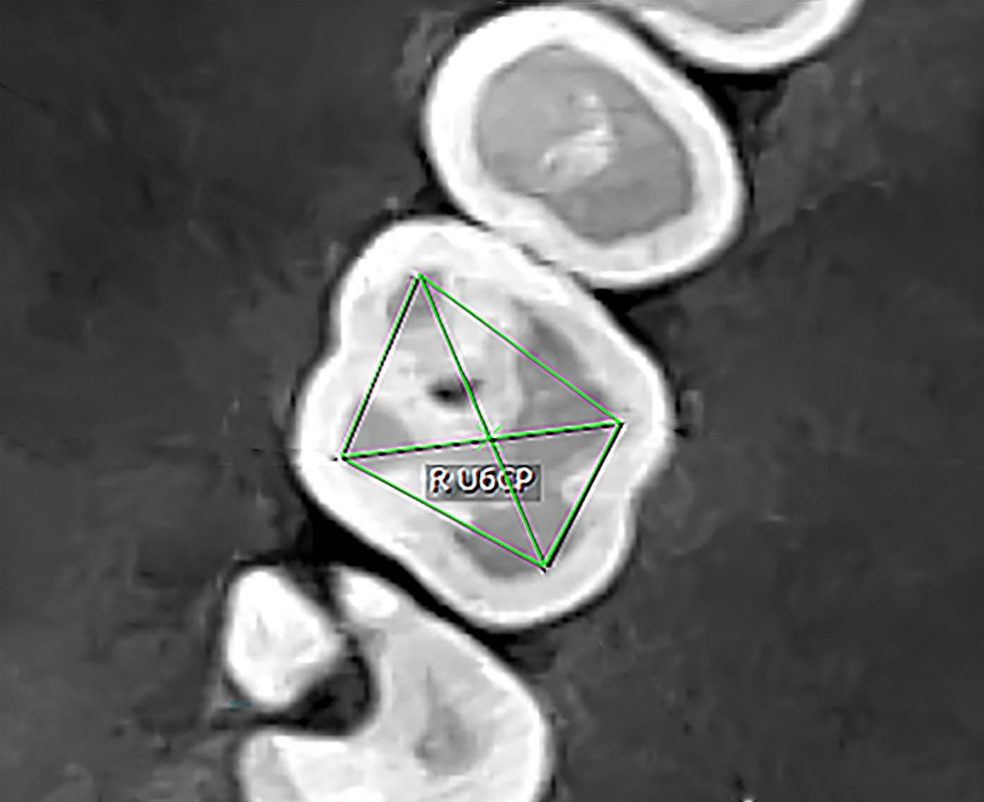
Determination of the crown point. R U6CP: right upper first molar crown point

The crown point was determined through the following: (1) in the sagittal plane, moving the line that represents the axial axis until it passes through the four cusps (Figure 1), and (2) in the occlusal plane, where four points representing the four cusps were determined to be closest to the inner edge of the enamel thickness, connecting the four points to create a quadrilateral geometric shape. The crown point is the point resulting from connecting the diagonals of the geometric shape (Figure 2).

**Figure 3:**
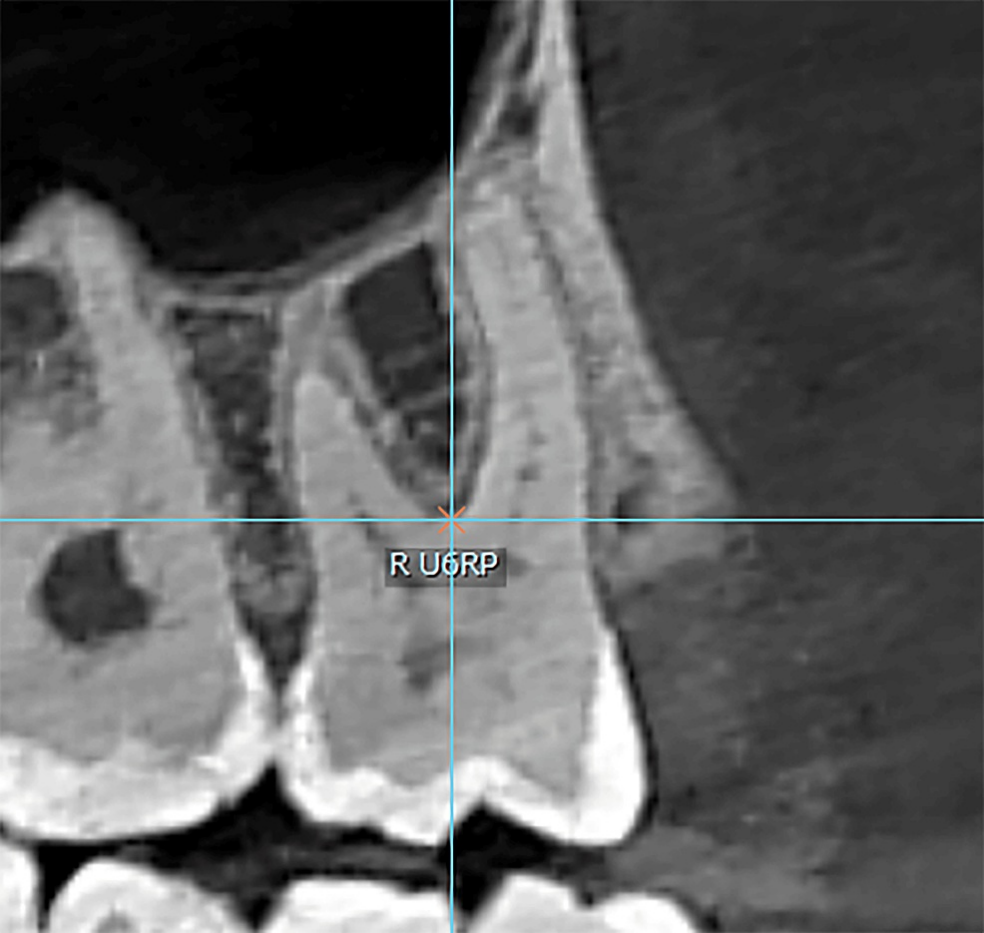
The axial axis passes through the molar bifurcation. R U6CP: right upper first molar root point

**Figure 4:**
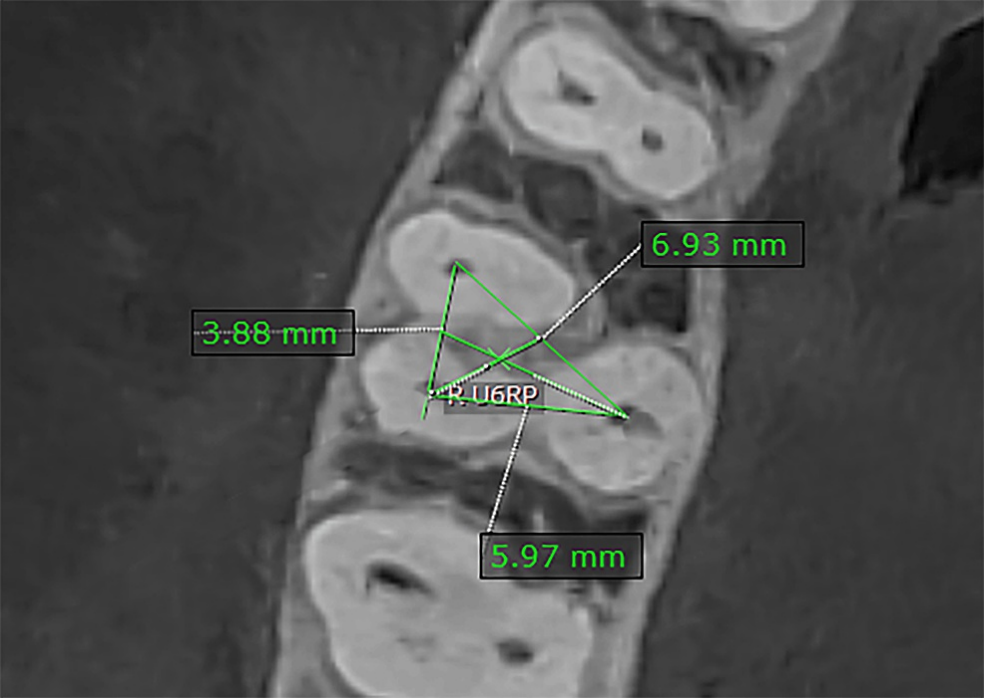
Determination of the root point. R U6CP: right upper first molar root point

The root point was determined through the following: (1) in the sagittal plane, moving the line that represents the axial axis until it passes through the bifurcation of the roots (Figure 3), and (2) in the occlusal plane, identifying three or four points representing the entrances to the root canals and connecting the three or four points to create a triangular or quadrilateral geometric shape. The root point is the point resulting from connecting the diagonals of a geometric figure (or the center of the triangle if there are three root canals) (Figure 4).

**Figure 5:**
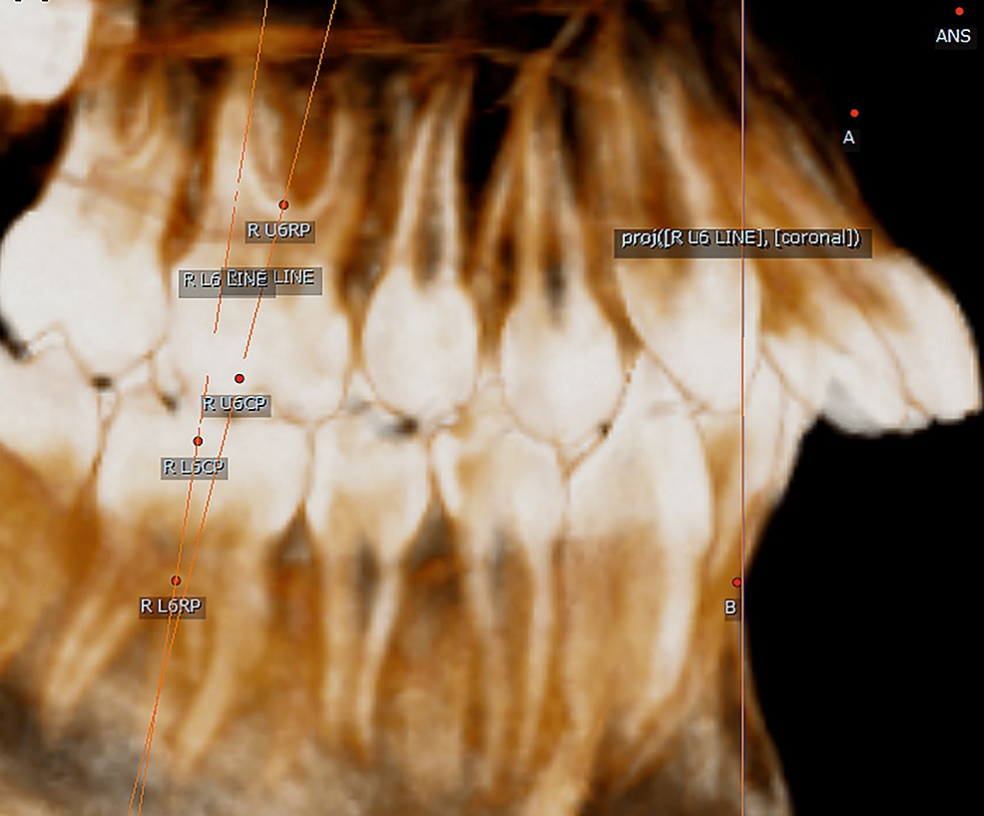
Molar axes. R L6CP: right lower first molar crown point; R L6RP: right lower first molar root point; R U6CP: right upper first molar crown point; R U6RP: right upper first molar root point; R L6 line: the line connecting (R L6CP) and (R LR6RP); R U6 line: the line connecting (R U6CP) and (R UR6RP)

The molar axis was established by the line connecting the crown point and the root point (Figure 5).

Reference line and molar inclination

The reference line was formed by the line connecting the right and left orbital points (or-or). The buccolingual inclination was measured relative to this reference line.

**Figure 6:**
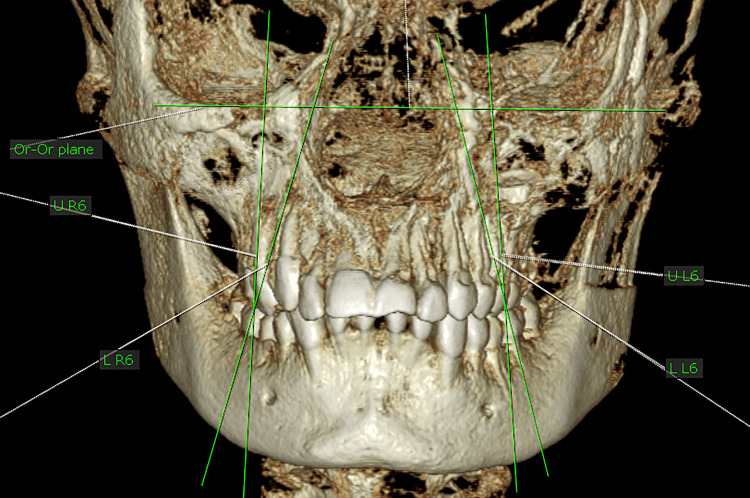
Measurement of the right and left maxillary and mandibular first molar buccolingual inclinations.

The value of the buccolingual inclination of the molar was measured through the following: (a) a projection was created for the molar axis at the frontal plane; (b) a projection of the or-or line was created at the frontal plane; and (c) the angle formed by the projections of the previous two lines was measured and was taken as the value of the buccolingual inclination of the molar (Figure 6).

Statistical analysis

IBM SPSS Statistics for Windows, Version 28.0 (Released 2021; IBM Corp., Armonk, New York, United States) was used to conduct the statistical analyses. Descriptive statistics such as means and standard deviations were calculated. The Shapiro-Wilk test was conducted to evaluate the data's normality. All variables, with the exception of two (lower left 6 in the normodivergent group and upper left 6 in the hypodivergent group), were normally distributed. The independent samples t-test and Mann-Whitney U-test were conducted for group comparison. Statistical significance was evaluated at a p-value threshold of 0.05, corresponding to a 95% confidence interval.

One week following the initial measurements, a second set of measurements was obtained by the same researcher (B.M.). Intraexaminer reliability was assessed through the calculation of intraclass correlation coefficients, which were used as a measure of systematic error.

## Results

The range of the intraclass correlation coefficients was 0.867-0.945, demonstrating high inter-observer reliability during repeated measurements. Values of buccolingual inclination of the upper and lower molars are presented in Tables 3-8. Statistical analysis revealed no significant difference in buccolingual molar inclinations between the normodivergent and hyperdivergent groups (Tables 3, 4). Conversely, statistically significant differences were observed between the hypodivergent group and the other groups. These differences were evident in the upper right and left molars and the lower right molars (Tables 5-8).

**Table 3:** Values of buccolingual inclination and the results of the independent samples t-test to study the differences between the average angle of the first molar between the hyperdivergent and the normodivergent. Values are presented as number (N) or mean±standard deviation. U R6: upper right first molar; U L6: upper left first molar; L R6: lower right first molar P<0.05 was considered significant.

Tooth	Hyperdivergent	Normodivergent	Difference of averages	T-value	P-value
U R6	88.03°±6.67°	88.13°±4.69°	0.092273	0.053	0.958
U L6	88.20°±3.72°	88.59°±2.13°	0.386818	0.422	0.675
L R6	103.07°±5.69°	102.94°±2.75°	-0.133636	-0.099018	0.922

**Table 4:** Values of buccolingual inclination and the results of the Mann-Whitney U-test to study the differences between the average ranks of the first molar angle between the hyperdivergent and the normodivergent. Values are presented as number (N) or mean±standard deviation. L L6: lower left first molar P<0.05 was considered significant.

Tooth	Hyperdivergent	Normodivergent	Mann-Whitney U-value	Z-value	P-value
L L6	103.81°±4.62°	103.34°±5.12°	231.000000	-0.258244	0.796

**Table 5:** Values of buccolingual inclination and the results of the independent samples t-test to study the differences between the average angle of the first molar between the hypodivergent and the normodivergent. Values are presented as number (N) or mean±standard deviation. U R6: upper right first molar; L R6: lower right first molar P<0.05 was considered significant.

Tooth	Hypodivergent	Normodivergent	Difference of averages	T-value	P-value
U R6	92.98°±4.6°	88.13°±4.69°	4.851364	3.459049	0.001
L R6	99.15°±3.67°	102.94°±2.75°	-3.790909	-3.868687	0.000

**Table 6:** Values of buccolingual inclination and the results of the Mann-Whitney U-test to study the differences between the average ranks of the first molar angle between the hypodivergent and the normodivergent. Values are presented as number (N) or mean±standard deviation. U L6: upper left first molar; L L6: lower left first molar P<0.05 was considered significant.

Tooth	Hypodivergent	Normodivergent	Mann-Whitney U-value	Z-value	P-value
U L6	91.43°±3.76°	88.59°±2.13°	95.500000	-3.439346	0.001
L L6	102.60°±3.36°	103.34°±5.12°	199.500000	-0.997833	0.318

**Table 7:** Values of buccolingual inclination and the results of the independent samples t-test to study the differences between the average angle of the first molar between the hyperdivergent and the hypodivergent. Values are presented as number (N) or mean±standard deviation. U R6: upper right first molar; L R6: lower right first molar, L L6: lower left first molar P<0.05 was considered significant.

Tooth	Hyperdivergent	Hypodivergent	Difference of averages	T-value	P-value
U R6	88.03°±6.67°	92.98°±4.6°	4.94364	2.860	0.007
L R6	103.07°±5.69°	99.15°±3.67°	-3.92455	-2.714	0.010
L L6	103.81°±4.62°	102.60°±3.36°	-1.21227	-.993	0.326

**Table 8:** Values of buccolingual inclination and the results of the Mann-Whitney U-test to study the differences between the average ranks of the first molar angle between the hyperdivergent and the hypodivergent. Values are presented as number (N) or mean±standard deviation. U L6: upper left first molar P<0.05 was considered significant.

Tooth	Hyperdivergent	Hypodivergent	Mann-Whitney U-value	Z-value	P-value
U L6	88.20°±3.72°	91.43°±3.76°	117.000000	-2.934492	0.003

## Discussion

CBCT offers the advantage of visualizing the entire tooth, eliminating the uncertainties associated with dental casts. The current study included CBCT scans of patients with ages ranging from 18 to 40 years, as tooth inclination can undergo alterations during the growth phase. Gender was not considered a variable due to previous researches indicating its negligible impact on inclinations [3,4,6]. A novel approach was employed to determine the longitudinal axis of molars, aiming to create a three-dimensional axis, contrasting with the previously used two-dimensional longitudinal axis for a three-dimensional structure. The literature that is currently available includes studies that are notably inconsistent and heterogeneous due to the lack of systematic organization of the comparison groups' vertical and sagittal characteristics.

Studies investigating buccolingual inclination in facial patterns without specific consideration of sagittal classification have yielded mixed results. Janson et al. reported no statistically significant differences between hyperdivergent and hypodivergent individuals regarding mandibular posterior tooth inclination [1], consistent with the findings of Masumoto et al. [14]. However, maxillary molars exhibited a greater buccal inclination in hyperdivergent subjects, a finding consistent with the observations of Banari et al. and Janson et al. [1,16]. Conversely, investigations by Ross et al., Beugre-Kouassi et al., and Ferreira et al. failed to identify statistically significant differences in buccolingual inclination among various facial patterns [13,15,17]. According to Banari et al., hyperdivergent participants had a higher lingual inclination in mandibular molars than their hypodivergent and normodivergent counterparts [16].

The influence of sagittal skeletal discrepancy on molar inclinations has been extensively documented in the scientific literature [3,10-12]. Studies have predominantly focused on describing sagittal characteristics, neglecting the analysis of vertical relationships. Consequently, the current investigation aimed to delineate molar inclinations in class II division I subjects exhibiting hypodivergent, hyperdivergent, or normodivergent vertical patterns.

An analysis was conducted to evaluate first molar inclination in patients with hypo-, hyper-, or normodivergent class II division I malocclusions. The findings revealed notable discrepancies between the groups. Specifically, the upper right and left first molars exhibited a significantly increased buccal inclination in the hyperdivergent and normodivergent groups compared to the hypodivergent group. This observation aligns with the findings reported by Janson et al. [1]. However, the study's results diverge from those obtained by Beugre-Kouassi et al. and Ferreira et al. [15,17]. The observed discrepancies in molar inclination may be attributed to the inclusion of multiple sagittal skeletal types within the sample population in their studies. Moreover, this divergence may be explained by the wider dental arch in the hypodivergent group, necessitating buccal inclination of the mandibular molars and lingual inclination of upper molars for proper occlusion [25]. Furthermore, the present study contradicts the findings of Tsunori et al. [2], demonstrating a significantly greater lingual inclination of the right mandibular first molars in both normodivergent and hyperdivergent individuals compared to their hypodivergent counterparts. The observed disparity in outcomes may be attributed to the differential distribution of muscular tension in terms of magnitude and orientation between the right and left sides. Nishi et al. showed a difference between left and right temporalis activity in class II malocclusion [26]. Additionally, a preliminary study by Al Zubaidi et al. concluded that an abnormal function may exert a different pressure on parts of the mandible, which could result in various forms of dentoalveolar underdevelopment [27].

Alternatively, this phenomenon could be a consequence of variations in the thickness of cortical bone in the vicinity of the mandibular molars.

The present study and similar investigations hold future implications for implementing tailored spacer treatments based on skeletal type and vertical facial pattern. These studies provoke questions regarding the feasibility of buccolingual inclination using clear aligners [28], the efficacy of torque incorporated in pre-adjusted brackets in achieving physiological tooth positions relative to cortical bone [29], and the potential for buccolingual inclination modifications to induce horizontal relapse [30].

Armed with the knowledge of natural tooth inclinations, clinicians can manipulate these inclinations through appropriate and physiologically sound alignment procedures.

Limitations of the study

The limitations of this investigation include the exclusive evaluation of class II division I subjects. Future research should focus on the independent examination of sagittal and vertical characteristics to facilitate a more comprehensive understanding of dental alignment variations among normo-, hyper-, and hypodivergent individuals in class I, class II, and class III malocclusions and relate the molar inclination to the severity of the malocclusion.

## Conclusions

No statistically significant difference was found between the hyperdivergent and normodivergent groups in terms of buccolingual molar inclinations. Notwithstanding, a marked buccal inclination was observed in the right and left upper first molars of both hyperdivergent and normodivergent subjects, contrasting with the hypodivergent group. Similarly, right lower first molars in the normodivergent and hyperdivergent groups exhibited a pronounced lingual inclination in comparison to their counterparts in the hypodivergent group.
